# Effects and mechanism of platelet-rich plasma on military drill injury: a review

**DOI:** 10.1186/s40779-020-00285-1

**Published:** 2020-11-22

**Authors:** Peng-Cheng Xu, Min Xuan, Biao Cheng

**Affiliations:** Department of Burn and Plastic Surgery, General Hospital of Southern Theater Command of Chinese PLA, No. 111 Liuhua Road, Yuexiu District, Guangzhou, 510010 Guangdong Province China

**Keywords:** Military drill injury, Platelet- rich plasma (PRP), Plantar fasciitis, Stress fracture, Rehabilitation

## Abstract

Military drill injury is a significant part of military medical research. The increase of training intensity and changes in training methods lead to differences in injury types. The ideal therapeutic modality should allow rapid healing at a reasonable cost and minimize impact on patients’ life. Platelet -rich plasma (PRP), a platelet concentrate, is rich in a variety of growth factors and widely used clinically as a minimally invasive treatment. It plays an important role in injury repair and rehabilitation. In this article, we review the therapeutic role of PRP in military drill injury and its possible underlying mechanisms, with a focus on plantar fasciitis, stress fractures and other common injuries, in order to provide basic support for military reserve.

## Background

Nowadays, military drill has become the main task of military work in the peace era. Strengthening actual combat military drill and improving the quality and level of troop training and preparation have become a new situation for army development, aiming at maintaining and improving troop combat readiness and effectiveness [1, 2].

With continuous intensification of military drill, the incidence of fatigue injuries, bone and joint injuries, ligament and other soft tissue injuries have gradually increased, leading to an increase in soldier disability rate and a decrease in attendance rate, which is very detrimental to the improvement of military force. It is considered that military drill injury has become a major threat to military reserve force [3]. Consequently, it is very necessary to find safer, more effective, and minimally invasive rehabilitation treatments to improve soldiers’ physical fitness and combat effectiveness.

Platelet -rich plasma (PRP) is a platelet concentrate, consisting of a large number of growth factors and effective components [4], which can improve local microenvironment and promote tissue regeneration and repair [5, 6]. Recently, PRP has been widely used in clinical practice such as orthopedics [7], dermatology [8] and plastic surgery [9]. As a minimally invasive treatment, it has achieved good results in promoting wound healing, relieving the pain of osteoarthritis and providing some functional benefits. Basic and clinical studies have found that PRP has a good application prospect in the treatment of injuries. Therefore, in this article, we will review the possible role of PRP in the treatment of military drill injury.

## Characteristics and application of PRP

In 1954, Kingsley CS proposed the concept of PRP in *Nature*, and subsequently, the function of platelet-derived growth factors and their clinical value were confirmed. PRP is a platelet concentrate extracted from autogenous blood by centrifugation and is categorized as a minimally manipulated tissue by the USA Food and Drug Administration (FDA) [10]. The platelet content in PRP is 3 to 8 times that of normal blood, and PRP also contains a small amount of other effective components such as white blood cells and fibrins [11]. When activated, platelets can release a large number of active factors associated with tissue regeneration [12]. It is confirmed that the proportion of growth factors in PRP is similar to that of the normal body. The synergistic effect of multiple growth factors plays an important role in tissue repair, participating in matrix formation, osteogenesis, and collagen synthesis (Table 1).

**Table 1:** The role of growth factors contained in PRP in tissue repair

Growth factors	Receptor	Role in tissue repair	References
HGF	c-Met	Stimulation of multiple cell growth, such as Hepatocytes, endothelial cells, epithelial cells	[13–16]
VEGF	VEGFR1(Flt1), VEGFR2(KDR/Flk1) VEGFR3(Flk4)	Stimulation of vascular endothelial cell proliferation and migration, initiation of angiogenesis response	[17]
TGF-β	TGF-β receptor I TGF-β receptor II	Involvement of inflammatory response, promotio of extracellular matrix secretion, stimulation of bone matrix deposition, inhibition of osteoclast formation and bone absorption	[18, 19]
IGF	IGF-IR, IGF-IIR	Chemotaxis of fibroblasts, promotion of cartilage and bone matrix formation, stimulation of ESCs proliferation and differentiation	[20–23]
EGF	EGFR (ERBB1)	Promotion of re-epithelization and participating in angiogenesis	[23, 24]
FGFs	FGFR1, FGFR2, FGFR3	Mitotic stimulation of articular chondrocytes, promotion of wound healing	[25, 26]
PDGF	PDGFRα, PDGFRβ (αα, αβ, ββ)	Promotion of angiogenesis and activation of macrophages, Stimulation of fibroblasts, vascular smooth muscle cells and Schwann cells proliferation and activity	[27–29]

*HGF* Hepatocyte growth factor, *VEGF* Vascular endothelial growth factor, *TGF-β* Transforming growth factor-β, *IGF* Insulin-like growth factor, *EGF* Epidermal growth factor, *FGF* Fibroblast growth factor, *PDGF* Platelet-derived growth factor

**Fig. 1:**
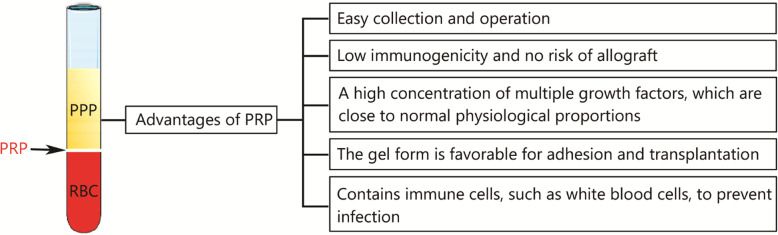
The advantages of PRP. PRP. Platelet-rich plasma; PPP. Platelet-poor plasma; RBC. Red blood cells

PRP has many advantages (Fig. 1), so it is widely used in treatment. Investigators found that the local application of mesenchymal stem cells associated with PRP in diabetic chronic ulcers is a well-tolerated therapy and could reduce ulcer size [30]. Multiple injections of PRP could improve joint function, reduce local pain and synovial inflammation in osteoarthritis patients [31, 32]. For rotator cuff tears, local injection of PRP could reduce rotator cuff pain and improve the function of the rotator cuff, which is conducive to subsequent rehabilitation treatment [33]. Meanwhile, PRP contains white blood cells, which could significantly enhance the body’s ability to remove local pathogens and resist infection [34]. Platelet-rich gels could also be used as an ideal scaffold for bone defect repair and help improve the postoperative healing microenvironment [35].

PRP has demonstrated good efficacy in a variety of conditions in orthopedics, sports medicine, oral and maxillofacial surgery, burn and plastic surgery and other fields; especially as a minimally invasive approach, it could become a promising new treatment for injury, including military drill injury (Table 2).

**Table 2:** The applications of PRP

Type of injuries	Functional effects	References
Bone and cartilage damage	Combination of bone graft to promote bone regeneration, Promotion of the differentiation of transplanted cells into chondrocytes	[36–39]
Sports injuries	Improvement of tendon healing quality and joint mobility, promotion of ligament injury repair, shortening recovery time of muscle strain, rehabilitation treatment of chronic sports injury	[40, 41]
Acute and chronic wounds	Promotion of angiogenesis granulation tissue formation, and re-epithelialization	[42–44]
Burn wound	Promotion of burn wound repair	[45]
Oral and maxillofacial injuries	Promotion of Alveolar bone regeneration, periodontal bone defect repair, and maxillary sinus filling	[46–48]
Fat transplantation	Improvement of transplanted adipose tissue quality and promotion of transplant survival	[49–51]
Others	Promotion of facial nerve, corneal injury repair, facial rejuvenation and hair regeneration, etc	[52–56]

## Effects of PRP on military drill injury

Military drill injury is a general term for a class of traumatic diseases with pathophysiological changes in military drill. According to the *Diagnostic Criteria and Principles of Prevention and Treatment of Military Drill Injury* [57] *and Notice on Carrying out the Monitoring and Reporting of Military Drill Injury*, military drill injury can be divided into five categories (Table 3). Among these, soft tissue and bone and joint injuries are the most common of all injuries. Therefore, based on the biological characteristics of PRP, we describe in detail the possible effects of PRP on these injuries, mainly introducing its effects on stress fractures, plantar fasciitis and rehabilitation treatment.

**Table 3:** The categories of military drill injuries

Category	Contents
Soft tissue injury	Injuries of muscles, ligaments, tendons, fascia, synovium, etc.
Bone and joint injuries	Acute fracture, stress fracture, joint dislocation, joint swelling, etc.
Organ injuries	Functional and structural damage of corresponding organs
Special environmental injuries	Heatstroke, frostbite, acute altitude sickness, etc.
Others	Chemical poisoning, burns, bites, and stings, etc.

### PRP and stress fractures

Stress fracture, also known as fatigue fracture, is a bone injury caused by excessive stress under the yield strength threshold. It is a common injury in military drill, athlete training, and special work, and most often occurs in the tibia, metatarsal, and femur [58]. Increasing intensity and difficulty of military drill drive a rising incidence of stress fractures.

The symptoms of stress fractures are occult, and the rate of missed diagnosis is high after fracture. Stress fracture readily recurs under external force, resulting in delayed healing, non-healing, and even osteonecrosis [58, 59]. Therefore, early diagnosis and recovery of bone function are critical to prognosis.

At present, for the treatments of stress fractures, brace, physical therapy, and local application of traditional Chinese medicine, which is good for blood circulation, are used [60]. However, as these treatments cannot penetrate the injury site, the therapeutic effect is often unstable. Compared with traditional therapy, minimally invasive treatment with no additional injury caused by surgery has attracted more attention.

Multiple studies in vivo and in vitro have proved that PRP can promote bone healing by the effects of multiple growth factors, with satisfactory clinical efficacy. The use of PRP combined with autogenous bone graft could enhance the quantity of bone formed, and its application may be advantageous to segmental mandibular defects [61]. Another study used PRP to treat mandibular fractures and found that injecting PRP along the fracture lines could enhance bone regeneration [62]. Meanwhile, basic research of rabbit fracture injury confirmed that intra-articular injection of autologous PRP combined with autologous adipose tissue-derived stem cells can help improve repair of cartilage defects, and the effect is better than that of surgical treatment alone [63].

In addition, some studies have shown that the mechanism whereby PRP promotes bone healing may be that platelets are involved in early response to bone injury and initiate the repair response by releasing growth factors, such as platelet derived growth factor (PDGF), insulin-like growth factor (IGF) and transforming growth factor-β (TGF-β) [64, 65]. The healing process of stress fractures also involves the above growth factors, which provides a theoretical basis for the application of PRP in stress fractures.

Although there is insufficient data on specific mechanisms and efficacy of PRP injection in the treatment of refractory fractures, its application prospect and value in promoting fracture healing, and bone defect reconstruction can be foreseen. The authors believe that PRP can be used as a conventional treatment for military drill-associated stress fractures.

### PRP and plantar fasciitis

Plantar fasciitis is a chronic degenerative change caused by prolonged standing or walking and overuse of the plantar aponeurosis and characterized by persistent tenderness in the lower part of the calcaneus tuberosity [66]. The characteristics of long and intensive military drill determine the prevalence of plantar fasciitis among soldiers.

Plantar fasciitis, due to degenerative changes, may become chronic, recurrent, and refractory. The ultimate goal of treatment is to relieve pain, restore fascia flexibility, and maintain foot motor function. Mild cases are mainly treated conservatively including foot orthoses, as well as foot traction training, with an effective rate up to 90% [67]. However, conservative treatment requires a long period of absolute bed rest, which often affects the normal life and work of soldiers. Consequently, the concept of minimally invasive treatment is put forward and widely adopted due to its convenience and accurate effect [68].

The most commonly used method of minimally invasive treatment is corticosteroid injection. The effect of hormone therapy is immediate, and improvement is good during follow up. However, hormone therapy may increase the risk of plantar fascia rupture, fat pad necrosis, skin atrophy, and infection, and is used on a short term basis, which limits its clinical application [69]. As a result, many investigators have applied PRP for minimally invasive treatment of plantar fasciitis. In a study of 25 patients with chronic plantar fasciitis, a single injection of 5 ml PRP using a 22 needle reduced pain but did not impair the biomechanical function of the foot, and patients resumed normal recreational activities 4 weeks after treatment, suggesting that this regeneration procedure is safe and effective [70].

Moreover, many studies have compared the effects of PRP with hormone therapy. Compared with steroid injection, local injection of PRP is an effective treatment for chronic plantar fasciitis. A prospective double-blind randomized study of PRP versus steroids showed that local injection of PRP was an effective treatment option for chronic plantar fasciitis with a long-term beneficial effect [71]. Another study compared the efficacy of PRP and corticosteroids in 60 patients with plantar fasciitis. The patients were followed up for up to 6 months, and the final evaluation showed that PRP could alleviate pain and improve the functional status, the same as hormone therapy. But unlike steroids, PRP is significantly more effective than steroids at 12 months, indicating that PRP treatment is more durable than cortisone injection. Investigators believe that PRP has good clinical effects and can avoid side effects associated with corticosteroid treatment [72]. In addition, the investigators also compared the effects of PRP and platelet-poor plasma (PPP) in chronic plantar fasciitis and found that a single injection of PRP was as effective as PPP. At the 6-month follow-up, both treatments led to significant improvement. This study has further improved the application of plasma components in plantar fasciitis. The treatment of chronic plantar fasciitis cannot only be treated with PRP but also can achieve a good prognosis combined with PPP [73]. This is very beneficial for patients because of the reduced whole blood volume by replacing PRP with PPP.

Based on clinical observation and efficacy evaluation, it is considered that PRP may replace conventional therapy as a first-line treatment for plantar fasciitis. The growth factors and effector molecules can avoid side effects caused by hormones and gradually improve chronic degenerative changes. The authors believe that it is feasible to use PRP as a potential minimally invasive treatment for military drill-associated plantar fasciitis.

### PRP and rehabilitation training

Regardless of treatment of stress fractures or plantar fasciitis, the ultimate goal of injury repair is to restore function. Rehabilitation therapy is divided into sports therapy and physical therapy, which can achieve the purpose of functional recovery by regulating physiological mechanisms such as human nerves, body fluids, endocrine and immunity. It is generally believed that receiving standardized and systematic rehabilitation treatment can greatly improve the psychological and physiological health of patients [74, 75].

Military drill injuries, especially tendon injuries, are mostly chronic and overworked injuries, involving muscles, tendons, and joints. It is considered that standardized rehabilitation training can effectively reduce the functional degradation of chronic injuries and prevent recurrence, which is essential for the recovery of organ function after military drill injury. Therefore, continuous innovation of rehabilitation treatment is beneficial to the maintenance of military power.

In recent years, investigators have tried to combine PRP with rehabilitation therapy to achieve complementary effects and preliminary confirmation of the role of PRP in rehabilitation. The study by West China Hospital showed that PRP combined with conventional physical therapy could reduce shoulder pain and improve shoulder joint function, which was better than conventional physical therapy alone [76]. Another study showed that PRP injection could improve healing rates and functional outcomes in patients with rotator cuff tears [77]. These studies have confirmed that conventional physical therapy combined with PRP injection could effectively enhance the curative effects of traditional rehabilitation therapy.

These positive effects are thought to be associated with the release of various signalling molecules following platelet activation, as well as the inhibitory effect of PRP on pain-causing inflammatory molecules. These studies affirmed the value of PRP in rehabilitation treatment and laid a foundation for the application of PRP in the post-rehabilitation treatment of military drill injury.

## Application prospect of PRP in war trauma

Although world wars have become history, regional wars are not rare. Compared with military drill injury, war traumas, such as ordinary firearm injuries, blast injuries, fuel-air bomb injuries and high-tech weapons injuries, are more serious and have a poor prognosis.

Nevertheless, the essence of care of war trauma is still tissue repair and regeneration. Currently, it is not possible to apply PRP to war trauma, but the feasibility of PRP treatment of military drill injury in this review provides a possibility for the application of PRP in war trauma in the future. With the development of technology, PRP can be prepared in new formulations, such as PRP freeze-dried powder [64] and PRP wafer [78], or other formulations in the future, like PRP-loaded sterile dressing. Improvement of PRP formulation will render transport and long-term storage more convenient, and it is hopeful that PRP can be used as a trauma dressing in battlefield first aid. Although PRP treatment cannot solve critical injuries; as an auxiliary material or minimally invasive approach, it will play an important role in frontline first aid.

## Deficiencies and controversies in PRP application

In this review, we mentioned the clinical application value of PRP, including fracture treatment and rehabilitation, and propose that it be used as a potential auxiliary therapy for military training injuries. However, there is still debate about the advantages of using PRP.

### The necessity of randomized controlled trials (RCT) in PRP studies

In the past decade, there has been a significant increase in the use of PRP to promote bone regeneration and soft tissue maturation. However, the additional benefits of this new technology are controversial in the literature. Although we have previously mentioned that some studies have been reported on the role of PRP in promoting bone formation and maturation rate, others have not observed any improvement.

Some studies suggested that PRP has no obvious advantage in promoting regeneration on bone defect. A study of intrabony defects found that PRP alone and bone graft both enhanced periodontal regeneration; however, the results with bone graft were better than PRP [79]. Another study assessed the effects of leukocyte-platelet-rich fibrin (L-PRF) in different intraoral bone implantation procedures, and considered its therapeutic effect was controversial, which needs further research and standardized protocols for the preparation [80]. Therefore, high quality RCT is very necessary.

### There is still no consensus standard for quality control of PRP

At present, the preparation methods of PRP are diversified, resulting in differences in platelet concentration and growth factor release, as well as unstable clinical effects, which is not conducive to widespread clinical application.

Here, it is considered that more high-quality RCT with large sample sizes will be needed to confirm the potential efficacy of PRP. At the same time, it is important to emphasize the importance of quality control standards, including preparation methods and dosages, which are critical and necessary for the clinical application of PRP, as well as the treatment of military drill injury.

## Conclusion

In summary, although there are still many controversies, some clinical trials have also confirmed the application value of PRP in promoting repair, relieving pain and providing some functional benefits, such as osteoarthritis [81, 82], synovitis [83], and refractory wounds [84, 85]. In the future, PRP may be a potential auxiliary therapy for military drill injury.

## Data Availability

All data generated or analysed during this study are included in this published article.

## Abbreviations

PRP: Platelet -rich plasma
PPP: Platelet-poor plasma
MSCs: Mesenchymal stem cells
HGF: Hepatocyte growth factor
VEGF: Vascular endothelial growth factor
TGF-β: Transforming growth factor-β
IGF: Insulin-like growth factor
EGF: Epidermal growth factor
FGF: Fibroblast growth factor
PDGF: Platelet-derived growth factor
